# Emotion Understanding, Social Competence and School Achievement in Children from Primary School in Portugal

**DOI:** 10.3389/fpsyg.2017.01376

**Published:** 2017-08-15

**Authors:** Maria da Glória Franco, Maria J. Beja, Adelinda Candeias, Natalie Santos

**Affiliations:** ^1^Department of Psychology, Faculty of Arts and Humanities, University of Madeira Funchal, Portugal; ^2^Department of Psychology, School of Social Sciences, University of Évora Évora, Portugal

**Keywords:** emotion understanding, social emotional competence, school achievement, elementary school, structural equational model

## Abstract

This study analyzes the relationship between emotion understanding and school achievement in children of primary school, considering age, gender, fluid intelligence, mother’s educational level and social competence. In this study participated 406 children of primary school. The instruments used were the Test of Emotion Comprehension, Colored Progressive Matrices of Raven, Socially Action and Interpersonal Problem Solving Scale. The structural equation model showed the relationship between the emotion understanding and school performance depends on a mediator variable that in the context of the study was designated social competence. Age appear as an explanatory factor of the differences found, the mother’s educational level only predicts significantly social emotional competence, fluid intelligence is a predictor of emotion understanding, school achievement and social emotional competence. Regarding the influence of sex, emotional understanding does not emerge as a significant predictor of social emotional competence in girls or boys. Multiple relationships between the various factors associated with school achievement and social emotional competence are discussed as well as their implications in promoting child development and school success.

## Introduction

The importance emotions have, in understanding the learning process and in the manner how individuals successfully complete their learning, is a topic that has commenced many studies. Learning is a complex individual process, but also a social one. Social interaction is one of it’s key components, that allows cognitive growth thanks to the guided learning that takes place in the proximal development area in which subjects move (Bruner, 1970/1998, Vygotsky, 1934/2008). In social interactions in the classroom, Social competences provide positive relationships between the individual and other social agents involved (Del Prette and Del Prette, 2005; Elijah and Madeira, 2013).

In this socio-constructivist approach, Saarni (1990) defends that emotions play a central role in social interaction. By understanding his emotions and feelings, the subject can decide, based on them, the most adequate behavior for each context, contributing to a constructive social and personal interaction, adapting conduct to context and situations. This ability to express, identify, understand and regulate emotions, as well as the ability to understand others’ emotions and feelings, is what the author defines as emotional competence (Saarni, 2000).

The concept of emotional competence emerges associated to different processes and abilities, having present the multitude of concepts that are studied as analogs to this one, such as emotional knowledge, emotional comprehension, emotional intelligence and emotional regulation (Bridges et al., 2004; Garner, 2010; Franco and Santos, 2015; McCormick et al., 2015; Roazzi et al., 2015; Djambazova-Popordanoska, 2016), and also taking into account the number of measuring instruments used in its assessment, such as observation, self-assessment or hetero-assessment (Djambazova-Popordanoska, 2016). On the other hand, the most studied abilities can be grouped into three components: emotional expression, emotional regulation and emotion understanding (Denham et al., 2003; Denham, 2006). As Saarni (1990) states, when we speak of emotional competence, “We are talking about how [children] can respond emotionally, yet simultaneously and strategically apply their knowledge about emotions and their expression to relationships with others, so that they can negotiate interpersonal exchanges and regulate their emotional experiences” (p. 116).

Being one of emotional competence’s components, emotion understanding has been studied within this context, but also outside of it, which gives place to great conceptual inconsistency (Franco and Santos, 2015). Despite the great diversity of definitions, emotion understanding can be defined as a set of abilities, which include understanding the relationship between emotions and other mental states, the knowledge of emotion regulating strategies and the understanding of ambivalent emotional responses (Pons et al., 2004). In detail, nine abilities make up for emotion understanding during childhood: (I) Recognition of emotions on the basis of facial expression; (II) Understanding of external causes of emotions; (III) Understanding of desire-based emotions; (IV) of belief-based emotions; (V) Understanding the influence of a reminder on a present emotional state; (VI) Understanding of the regulation of an experienced emotion; (VII) Understanding of the possibility of hiding an underlying or true emotional state; (VIII) Understanding of mixed emotions; and (IX) Understanding of moral emotions (Pons et al., 2004). These abilities don’t all occur at the same age, but develop with age, starting with the most elementary abilities to the more complex. They coexist throughout development and makeup for each other’s pillars as new abilities are acquired. Research shows that with age there is a greater performance in each of the abilities (Pons and Harris, 2005; Santos, 2012; Silva, 2012). Cognitive development also influences the development of emotional understanding, facilitating the manifestation of new abilities (Albanese et al., 2010; De Stasio et al., 2014).

The development of emotion understanding goes through three phases: the external phase, the mental phase and the reflexive phase (Pons et al., 2004; Tenenbaum et al., 2004; Pons and Harris, 2005). During the external phase, between ages three and six, children are able to identify different emotional expressions, to understand external situational factors that may trigger emotions and understand that the recollection of a past event may trigger an emotional reaction, in other words, recognize emotions, understand the external causes, desires and recollections. The mental phase, between ages five and nine, embraces the fact that children understand that emotions depend on beliefs and awakened desires, and that not always the experienced emotions are the expressed ones, for there is a difference between apparent emotions and real emotions (e.g., a child can seem apparently calm because he is in a party of his best friend but inside he can be very sad because he lost is favorite toy). Lastly the reflexive phase, from ages eight and twelve, involves the ability to understand composite emotions, these are emotions that may occur simultaneously, that sometimes may be contradictory. The ability to understand that certain emotions are associated to moral values in their life context and the aptitude to regulate emotions in order to adapt them to that very same context, are also a characteristic of this phase.

Numerous studies have related emotional competence to school achievement, from preschool to higher education, not forgetting primary school. These studies are very heterogeneous, not only because of the diversity of the emotional competences that are approached (emotional knowledge, emotional regulation, emotional understanding, emotional intelligence), as well as because of the manner in which academic achievement is tested. Sometimes, through learning outcomes (final grades of the school year or of specific subjects, most commonly mathematics and the mother tongue), other times through adjustment skills or adaption to school context. Despite the dispersion of studies, that disturbs the true understanding of the connection between these two variables, as we will later come across, their contributions indicate the existence of a relation between these two variables, either directly or mediated by different variables.

The research on the influence of emotional competence in school achievement in primary education is scarce, but the review of the existing studies regarding preschool education is a relative contribution in two aspects, understanding the importance that development of these competences has in attending primary school successfully: (a) in the preparation of certain cognitive skills for school learning; (b) the development of the necessary requirements for transitioning school level. Note the studies on emotion understanding that demonstrate it is related to important cognitive processes that affect learning, such as language competence (Cutting and Dunn, 1999; Colwell and Hart, 2006), attention processes among them, focusing and sustaining attention in a classroom capacity (Nelson et al., 1999).

The research on emotional regulation proof it’s relation to “readiness to learn,” “teachability” (Denham, 2006), “school readiness” (Raver, 2002, 2003; Raver and Knitzer, 2002), productivity in the classroom (Graziano et al., 2007), success in mathematics and reading ability (Blair and Razza, 2007; Graziano et al., 2007), which is also mediated by factors such as attention (Izard et al., 2001; Trentacosta and Izard, 2007) and behavioral self-regulation (Howse et al., 2003). Regarding school adjustment, both in the research on emotion understanding (Shields et al., 2001) as well as in the studies on emotional regulation (Denham et al., 2012b, 2014; Herndon et al., 2013) there is a positive relation with the first. Research describes the implementation socio-emotional competence development programs in preschool (e.g., SEL Programs, Curriculum PATH) and also reveals that the participation in these programs, with consequent improvement in these skills, leads the individual to better his capacity to adapt to school and is predictive of greater academic results (Bierman et al., 2008; Denham and Brown, 2010; Denham et al., 2012b, 2014; Low et al., 2015).

Rhoades et al. (2011) developmental study of the transition to primary school demonstrates the impact that promotion of emotional competences has in primary school. These authors followed 341 disadvantaged children from public schools in a district of Northeastern United States, for 3 years, from preschool to first grade, that were submitted to an emotional competence program in preschool, under the Head Start program, and tried to understand through the analysis of structural equations, how emotion knowledge is related to attention skills and academic competence. The results may be summarized in the following way: (a) preschool emotion knowledge is a significant predictor of later academic achievement; (b) attention skills are one mediator of this relationship; (c) children’s emotion knowledge and attention skills are two key components for improving academic competence in the early school years.

There is very little research in this area at Primary School level. Trentacosta et al. (2006) showed that: (a) emotion knowledge predicted attentional competence while controlling for age, gender, verbal ability, and initial levels of attentional competence; (b) Emotion knowledge also mediated the relation between verbal ability and attentional competence; (c) Emotion knowledge may provide children with the satisfactory peer and teacher relations that foster achievement motivation and attention to academic tasks to a level commensurate with their general cognitive ability. The study of McKown et al. (2016), which used structural equation models, supports the relation between socio-emotional skills and academic outcomes. It also shows that the relation between socio-emotional skills and reading is measured by social behavior (socially skilled behavior). But the measuring of social behaviors, in the relation between socio-emotional competences and the grades in mathematics, are not demonstrated, for they only appear in one of the studied samples.

In Portugal we found two studies that relate emotion understanding to academic achievement in elementary school (Silva, 2012; Rocha, 2016). Both studies use as an instrument the Test of Emotional Comprehension- TEC (Pons and Harris, 2000; Pons et al., 2004; Rocha et al., 2015) and measure Academic Achievement through grades obtained in Portuguese and Mathematics. In both studies (Silva, 2012; Rocha, 2016), the results suggest some relation between emotion understanding and the grades obtained in Portuguese and Mathematics. However, and according to the studies carried out by Rocha (2016) emotion understanding does not directly predict academic achievement, but this influence is measured by social competence. On the contrary, social competence is a direct predictor of school achievement.

Various models have been developed to explain the link between emotional competences, social competences and academic achievement. Eisenberg et al. (2005) suggest that children’s emotional regulation (influenced by language skills as well as emotion knowledge and emotional understanding) affects their academic achievement, not only directly, but also through the measure of social competences. These authors also consider that there may be direct effects of emotion understanding and language skills on academic achievement and social competence. This model is sustained on research that approaches social competence, demonstrating that children that apply their emotion understanding in emotionally charged situations have better relationships with peers: these students are more prosocial, are considered more socially skilled by the teachers and are identified as being more pleasant by peers. This data has been confirmed in longitudinal studies (Izard et al., 2001; Denham et al., 2003, 2013; Ensor et al., 2011).

Various researchers have demonstrated the validity of this model, including the influence of other variables such as age, gender, parents’ academic level and socioeconomic level. Montroy et al. (2014) confirmed, through the analysis of structural equations, that the relation between children’s self-regulation in preschool and their reading ability, was measured by social functioning, social competences and behavioral problems, and that this relation was similar in both boys and girls. Valiente et al. (2011) confirmed, in a longitudinal study, that there is an effect of emotional regulating competences on academic achievement in children, when measured in ages from 4 to 8, which is later mediated by social competences, when measured in the same children 2 years later (6 and 8 years old). The socioeconomic level, age and gender affect academic achievement, but only children’s socioeconomic level affected their regulating competences. Neither these competences, nor children’s social competences, are affected by gender or age.

Nonetheless, studies by (Denham et al., 2012a,b, 2013) confirm the link between emotion understanding and academic achievement, mediated by social competences in preschool children. These authors also determined that preschool children’s emotion understanding was a predictor of the perception teachers had of their social competences and academic adjustment. In these studies, a child’s age, the mother’s academic level and the child’s self-regulating ability presented effects on emotion understanding: emotion understanding was greater in older children, with higher levels of self-regulation and with mothers who had higher academic levels.

In the research carried out by Rocha (2016) the link between emotion understanding and academic achievement, facilitated by social competence, in children of the 3rd and 4th grade of school, was confirmed. The mothers’ academic level was a predictor of their social competence and their academic achievement, but not of their emotion understanding, age and gender not having a significant influence on the model.

The present study aims to further study the model presented by Rocha (2016) using a more comprehensive model and including fluid intelligence, following de Spearman’s theory of cognitive capacity (*g* factor, Simões, 1995; McCallum et al., 2001), since cognitive skills are considered better predictors of academic achievement (Colom and Flores-Mendoza, 2007; Di Fabio and Palazzeschi, 2009, 2015; Downey et al., 2014).

Therefore, our objective is to determine the relation between fluid intelligence, mother’s educational level, age, gender, emotion understanding and social competence as predictors of children’s academic achievement in the first 4 years of primary school. The following hypothesis were defined:

H1: Children’s emotion understanding predicts school achievement
H2: Social competence is a mediating factor in the relationship between emotion understanding and school achievement
H3: Children’s fluid intelligence predicts children’s emotion understanding, social competence and school achievement
H4: Children’s age predicts emotion understanding and social competence
H5: Mother’s educational level predicts children’s emotion understanding, social competence and school achievement
H6: Children’s gender predicts differently the structural model

**FIGURE 1:**
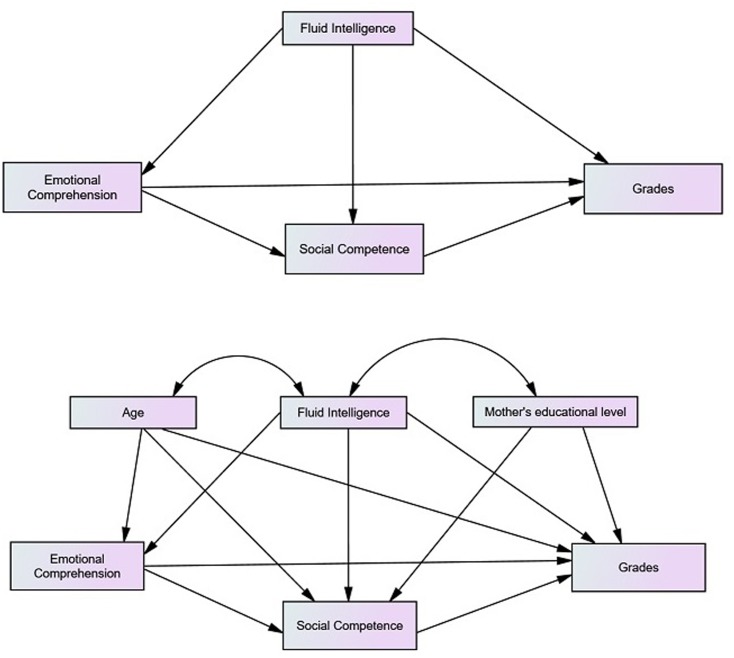
Proposed model of predictors of school achievement.

As a starting point to test our hypothesis and being based on various studies, the different relations to be established were synthesized in **Figure 1**. The relations were tested through the analysis of Structural Equations.

## Materials and Methods

### Participants

Data collection was carried out in four primary schools of the Autonomous Region of Madeira, which resulted in a sample of 406 students, 196 female and 210 male, aged between 6 and 11 (*M* = 7.93, *SD* = l. 43) from grades 1st to 4th.

Several parents and six teachers, of six different classes (one 1st grade class, two 2nd grade classes, two 3rd grade classes and one 4th grade class) of one of the schools participated in the study. Teachers assessed the social behavior of 108 students (26.61% of the sample), ages ranged between 6 and 11 (*M* = 7.63, *SD* = 1.12). The parents of 86 children (20.44% of the sample) assessed the social behavior of their children, whose ages ranged between 6 and 10 years (*M* = 7.58, *SD* = l.09). In **Table 1** the characteristics of different samples are presented in greater detail.

**Table 1:** Participants description.

Variable	Level	Participants (Total) (*n* = 406)		Participants assess by teachers (*n* = 108)		Participants assess by parents (*n* = 86)	
				
		*f*	%	*f*	%	*f*	%
Gender	Females	196	48.3	56	51.9	49	57.0
	Males	210	51.7	52	48.01	37	43.0
School year	1st year	104	25.6	15	13.9	12	14.0
	2nd year	100	24.6	33	30.6	25	29.1
	3rd year	82	20.2	38	35.2	32	37.2
	4th year	120	29.6	22	20.4	17	19.8
Mother’s educational level	1st cicle^a^	52	16.3	12	13.2	8	11.1
	2nd Cicle^b^	95	29.8	19	20.9	17	23.6
	3rd cicle^c^	77	24.1	32	35.2	24	33.3
	High school^d^	70	21.9	28	20.9	19	26.4
	Higher education^e^	25	7.8	91	6.6	4	5.6


*^a^First four grades of Portuguese educational system; ^b^5th and 6th grades of Portuguese educational system; ^c^7th to 9th grade of Portuguese educational system; ^d^10th to 12th grade of Portuguese educational system; ^e^University education.*

### Instruments

To assess the student’s performance on emotional competence, the computerized Portuguese version translated by Roazzi et al. (2008) of the Test of Emotion Comprehension (TEC), of Pons et al. (2004), was used. This instrument assesses the level of nine components of emotional understanding, where a point is attributed to each correctly answered component. Each child can obtain a minimum of zero and a maximum of nine points. Using the Kuder–Richardson coefficient as a measure of reliability, acceptable levels of internal consistency have been found (KR-20- = 0.72).

To assess the students’ social competence were used two different measures: (a) Social Competence Assessment Scales [SCAS (7-11) by Candeias, 2008]; (b) Problem solving in social situations index (PRI) of the Social Intelligence Cognitive Test (PCIS, Candeias, 2007). The SCAS is a 360-degree instrument that evaluates, from the perspective of students, parents and teachers, the degree of competence and the difficulty level facing five different social situations, through a three-point Likert scale. Each child can obtain a minimum of 10 points and a maximum of 30 points. In this study, adequate internal consistency levels were found, both in students’ scales (α = 0.73) as well as in parents’ scales (α = 0.87) and teachers’ scales (α = 0.97). The PCIS is an ability instrument and measures several skills involved in the resolution of social problems, regarding the quality of the components of the process (process dimension) as well as the level of interpersonal knowledge that the individual displays (contents dimension). Facing three different images containing social situations, the child has to identify the social problem, the best solution to it and the best strategies to achieve it. In this index, each child may obtain a minimum of 0 points and a maximum of 63 points. Adequate levels of internal consistency were found (α = 0.86).

In order to evaluate the students’ fluid intelligence, the adapted Portuguese version of the Colored Progressive Matrices (CPM, Simões, 2000) was used.

To assess school achievement, the average marks in the following subjects, Portuguese and mathematics, were calculated at the end of the semester where the emotional and social competence tests were applied.

The school grades go according to the following scale: 1 – unsatisfactory, 2 – marginally satisfactory, 3 – satisfactory, 4 – very satisfactory, 5 – very good or excellent.

### Procedure

Data collection took place at the school attended by the children, with the required written consent and authorization granted by the parents, school board as well as the children, following the ethical principles of scientific research. In order to ensure that reading skills did not affect the results, experienced psychologists applied the assessment scales, individually, reading each question, taking between 30 and 45 min. The instruments were applied in the following order: TEC, CPM, SACS and PRI. The questionnaires were handed to the teachers in a closed envelope and were completed and returned in the time space of 1 week. Parents completed the questionnaires during a parents meeting that took place to explain the objectives of the study.

### Data Analysis

Following collection, data was input to the SPSS software, version 23.0 for Windows. Descriptive statistics and Pearson correlation coefficients were calculated, along with structural equation analysis of the causal model of school achievement. It was evaluated with the AMOS software (version 23.0) as described by Maroco (2014) using the maximum likelihood model, the most adequate for small samples (200–500 subjects). In an adjustment to the model, a two-step strategy was used: in the 1st step, the measurement model was adjusted and in the 2nd step, the structural model was adjusted.

Due to the presence of missing in the sample, the averages and intersections were estimated. Data analysis took place to reveal the presence of cases of univariate and multivariate outliers. No participants with outlier values were found. The normality of the variables was determined by the measures of skewness (*sk*) and univariate Kurtosis (*ku*), presented in **Table 2**. None of the variables presented values of *sk* or *Ku* that were indicators of severe violations to normal distribution (|*sk*| < 3 and |*ku*| < 7–10, refer to Maroco, 2014).

**Table 2:** Correlation matrix and descriptive statistics of variables collected.

	1	2	3	4	5	6	7	8	9
(1) Age	1								
(2) Mother’s educational level	-0.10	1							
(3) Problem Resolution Index (PRI)	0.23**	-0.03	1						
(4) Social Competence Assessment Scale – Self (SCAS-S)	-0.24 **	0.02	0.00	1					
(5) Social Competence Assessment Scale – Parents (SCAS-P)	0.02	-0.05	0.11	0.05	1				
(6) Social Competence Assessment Scale – Teachers (SCAS-T)	0.05	0.14	0.27**	0.00	0.49**	1			
(7) Test of Emotional Comprehension	0.42**	0.07	0.21**	-0.14**	0.06	0.32**	1		
(8) Raven Colored Progressive Matrices (CPM)	0.42**	0.16**	0.28**	-0.17**	0.11	0.27**	0.46**	1	
Grades	-0.13**	0.31**	0.13**	0.10*	0.31**	0.64**	0.17**	0.30**	1
*n*	406	319	272	402	86	108	406	406	406
Mean	7.93	2.75	20.87	24.27	22.94	18.75	5.28	23.82	3.20
SD	1.43	1.19	7.44	3.85	4.27	7.09	1.68	6.04	1.05
Minimum	6	1	5.00	13.00	10.00	10.00	1.00	7.00	1.00
Maximum	11	5	49.00	30.00	30.00	30.00	9.00	36.00	5.00
Skewness	0.34	0.19	0.40	-0.33	-0.59	0.22	-0.16	-0.23	0.02
Kurtosis	-0.81	-0.93	0.31	-0.46	0.21	-1.29	-0.54	-0.59	-0.58


*^∗∗^Correlation is significant at the 0.01 level (two-tailed). ^∗^Correlation is significant at the 0.05 level (two-tailed).*

The quality of the adjustment of the model was determined according to quality adjustment indexes and respective reference values according to Maroco (2014) in which: *X*^2^/*df* < 2-3, CFI e TLI > 0.90. The RMSEA was also utilized, with a confidence interval (CI) of 90% and the probability of the RMSEA ≤ 0.05. A CI of 90% with an upper confidence bound less than 0.10 and *p*[RMSEA] ≥ 0.05 was considered a reasonable adjustment indicator, based on the belief that when the RMSEA value is less than 0.05, the adjustment is very good. In order to compare models, the AIC was used, being the best model, the one that presents lower values in this index. The quality of the local adjustment of the model was determined by factor weights and individual reliability of the items. The significance of the structural coefficients was evaluated with a *Z*-test produced by the AMOS software (Critial Ratio and *p*-value). The significance of the direct, indirect and total effects was determined by the Sobel test, as described by Maroco (2014).

Lastly, to test gender differences in the final model, path analysis with the same predictors and trajectories was done on both boys and girls. The invariance of the measuring model was tested in both groups, by comparison of the random model (with factorial weights and variances/covariances of the arbitrary factors) with the constrict model, where factorial weights and the variances and covariances of both groups were set. Lastly, the invariance of the structural model was determined by comparison of the model with free structural coefficients versus the model with fixed structural coefficients and equal in both groups. The statistical significance of the difference between both models was verified by the Chi-square test.

## Results

### Preliminary Analysis

The descriptive statistics and correlations between variables are presented on **Table 2**. Emotion understanding tested with TEC presents weak positive correlations with school marks (*p* = 0.001) and with the index of social problem solving (*p* = 0.001), indicating some relation between child’s emotion understanding, school achievements and the capacity to solve problems of social nature. Moderate positive correlations were also found with social competence, when evaluated by the teachers (*p* < 0.001) and with age (*p* < 0.001). On the opposite, poor negative correlations were found between emotion understanding and social competence when self-assessed by the children (SCAS-S) (*p* = 0.006), suggesting that better emotion awareness children have, the less positive their self-assessment will be regarding their social competences.

The connections between the instruments that assess social competence and school achievement were also significant and positive. These correlations are weak with the PRI (*p* = 0.028) and with the SCAS-S (*p* = 0.047), moderate with the SCAS when evaluated by the parents (SCAS-P) (*p* = 0.004) and strong with the SCAS when assessed by the teachers (SCAS-T) (*p* < 0.001). There are no correlations between the instruments that test social competence and the mother’s educational level. There are only poor correlations between age and the SCAS-S (*p* < 0.001) and the PRI (*p* < 0.001).

Moderate positive correlations were found between fluid intelligence, evaluated by Raven’s Colored Progressive Matrices, and children’s school marks (*p* < 0.001). Fluid intelligence is also related to emotion understanding (*p* < 0.001) and to social competence when assessed by the PRI (*p* < 0.001), by the SCAS-S (*p* = 0.001) and by the teachers SCAS (*p* = 0.004). Age (*p* < 0.001) and the mother’s educational level (*p* = 0.005) are also correlated to fluid intelligence.

Lastly, we found moderate positive correlations between school grades and mothers’ educational levels (*p* < 0.001) and poor negative correlations between school grades and the child’s age (*p* = 0.011).

### Measuring Model

Before testing our theoretic model, the adjustment indexes of the reference model were calculated, in order to determine if the instruments used (SACS in its three versions and PRI) could be grouped in one latent variable that describes student’s social competence. Although all adjustment indexes are adequate, (*X*^2^/*df* = 0.006; CFI = 1.000; TLI = 1.477; RMSEA = 0.000, IC at 90%[0.00;0.00], *p[RMSEA]* = 0.998; AIC = 24.012), the factorial weight (0.00) and reliability values (0.00) were very low in SACS, in the child’s self-assessment version. The results for this version of the SACS scale were excluded from the measuring model, because it doesn’t seem to saturate the social competence factor.

### Structural Model

According to Rocha (2016), a causal model of emotion understanding of academic achievement was evaluated, mediated by social competence. The fit indexes for each of the models are represented in **Table 3**.

**Table 3:** Fit indices of tested models.

Model	*X*^2^/*gl*	CFI	TLI	RMSEA			AIC
					
				RMSEA	90% CI	*p*	
Reference values	<0 2	>0.90	>0.90	<0.10	[;<0.10]	> 0.05	the lower
Model 1	3.302	0.987	0.874	0.075	[0.000;0.172]	0.211	29.302
Model 2	3.256	0.993	0.892	0.075	[0.000;0.171]	0.215	41.256
Model 3	2.207	0.965	0.894	0.055	[0.026;0.083]	0.356	90.489
Model 4	1.935	0.966	0.918	0.048	[0.020;0.074]	0.511	87.030


**N* = 406; endogenous variable: academic achievement; exogenous variables: model 1 – age, mother’s educational level, and CPM; model 2 – age, mother’s educational level, CPM, and TEC; model 3 and 4 – age, mother’s educational level, CPM, TEC and social competence.*

In model 1, the variables that consistently contribute to the explanation of our endogenous variable, academic achievement, were included as exogenous variables, age, mother’s educational level and fluid intelligence assessed by CPM.

The adjustment indexes of model 1 are low. All trajectories are significant, being the explained variance of academic achievement in this model 20.8%, the greatest predictor of fluid intelligence (**Table 4**). Age and academic level are significant predictors of academic achievement.

**Table 4:** Summary of covariate regression weights for structural equation models

Model	Direct effects			*b*	β	*p*
Model 1 (*R*^2^*_Grades_* = 0.208)	Grades	<–	CPM	0.065	0.0379	<0.001
	Grades	<–	Mother’s educational level	0.192	0.218	<0.001
	Grades	<–	Age	-0.198	-00.270	<0.001
Model 2 (*R*^2^*_Grades_* = 0.220; *R*^2^*_TEC_* = 0.281)	TEC	<–	CPM	0.093	0.335	<0.001
	TEC	<–	Mother’s educational level	0.060	0.042	0.382
	TEC	<–	Age	0.332	0.281	<0.001
	Grades	<–	TEC	0.079	0.127	0.014
	Grades	<–	CPM	0.058	0.337	<0.001
	Grades	<–	Mother’s educational level	0.187	0.213	<0.001
	Grades	<–	Age	-0.224	-0.306	<0.001
Model 3 (*R*^2^*_Grades_* = 0.526; *R*^2^*_TEC_* = 0.277; *R*^2^*_SocialC_* = 0.176)	TEC	<–	CPM	0.096	0.346	<0.001
	TEC	<–	Age	0.323	0.273	<0.001
	Social Competence	<–	TEC	1.160	0.285	0.002
	Social Competence	<–	CPM	0.245	0.217	0.021
	Social Competence	<–	Mother’s educational level	0.639	0.111	0.201
	Social Competence	<–	Age	-0.557	-0.116	0.197
	Grades	<–	TEC	-0.027	-0.044	0.531
	Grades	<–	CPM	0.035	0.203	0.003
	Grades	<–	Mother’s educational level	0.128	0.145	0.015
	Grades	<–	Social Competence	0.093	0.611	<0.001
	Grades	<–	Age	-0.172	-0.235	<0.001
Model 4 (*R*^2^*_Grades_* = 0.526; *R*^2^*_TEC_* = 0.277; *R*^2^*_SocialC_* = 0.146)	TEC	<–	CPM	0.096	0.346	<0.001
	TEC	<–	Age	0.323	0.273	<0.001
	Social Competence	<–	TEC	0.931	0.233	0.002
	Social Competence	<–	CPM	0.236	0.213	0.014
	Grades	<–	Social Competence	0.094	0.607	<0.001
	Grades	<–	CPM	0.035	0.204	0.002
	Grades	<–	Mother’s educational level	0.174	0.199	<0.001
	Grades	<–	Age	-0.220	-0.302	<0.001


In model 2, emotion understanding was included as a predictor of academic achievement, with direct trajectories for age, mother’s educational level and fluid intelligence as predictors of emotion understanding. Once again, the model presents minor adjustment indexes, explaining 22.0% of the variance. Fluid intelligence is the best predictor of academic achievement, as well as emotion understanding, followed by age. Mother’s educational level continues to be a predictor for academic achievement, along with emotion understanding as significant predictor too. Mother’s educational level is not a significant predictor of emotion understanding, excluding this trajectory therefore in the following models (**Table 4**).

**FIGURE 2:**
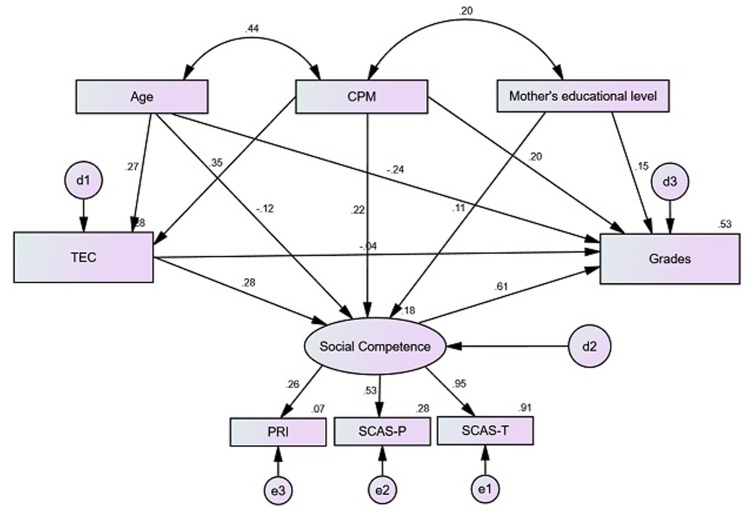
Structural model 3 of emotion understanding for academic achievement, mediated by social competence, with estimates for structural coefficients, factorials loads and standardized individual reliability (*X*^2^/*df* = 2.207, CFI = 0.965, RMSEA = 0.055, AIC = 90.489).

Finally, in model 3, represented in **Figure 2**, the complete model was tested, with direct trajectories of fluid intelligence and of age for emotion understanding; mother’s academic level, fluid intelligence and age for social competence; and of emotion understanding, mother’s educational level and fluid intelligence for academic achievement. Social competence was considered as a mediator, between emotion understanding and academic achievement.

**FIGURE 3:**
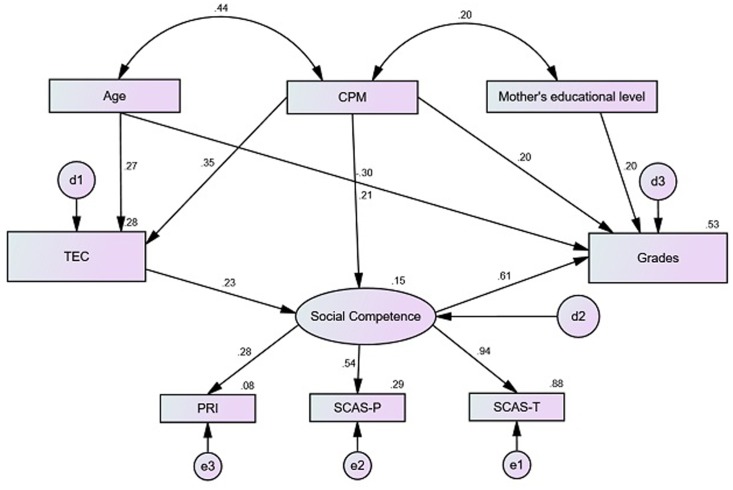
Structural model 4 of emotion understanding for academic achievement, mediated by social competence, with estimates for structural coefficients, factorials loads and standardized individual reliability (*X*^2^/*df* = 1.935, CFI = 0.966, RMSEA = 0.048, AIC = 87.030).

Model 3 presented adequate adjustment levels, explaining 52.6% of the variance regarding academic achievement. It was found that the trajectory for emotion understanding → academic achievement was no longer significant in this model, social competence becoming the mediator of the relationship between these two variables. We observed yet that mother’s educational level and age are not significant predictors of social competence. Therefore, these trajectories were removed from the final and 4th model, represented in **Figure 3**.

In model 4, the trajectories are significant (**Table 4**) demonstrating a bettering in the quality of the model comparatively to model 3, and maintaining the same percentage of variance regarding academic achievement (52.6%). It was also found that social competence has a very important direct effect in the explanation of emotion understanding. Emotion understanding presents only one indirect relation with academic achievement, mediated by social competence, and confirmed by the Sobel test (*b* = 0.014, β = 0.134, *Z* = 3.334, *p* = 0.001). Fluid intelligence determined by TEC (*b* = 0.069, β = 0.070, *Z* = 2.799, *p* = 0.005) presents significant indirect effects on social competence, and significant indirect effects on academic achievement through social competence (*b* = 0.016, β = 0.152, *Z* = 2.034, *p* = 0.042). The total standardized effects indicate that highest predictor is social competence (β = 0.591), followed by fluid intelligence (β = 0.356), mother’s educational level (β = 0.193) and emotion understanding (β = 0.134).

**FIGURE 4:**
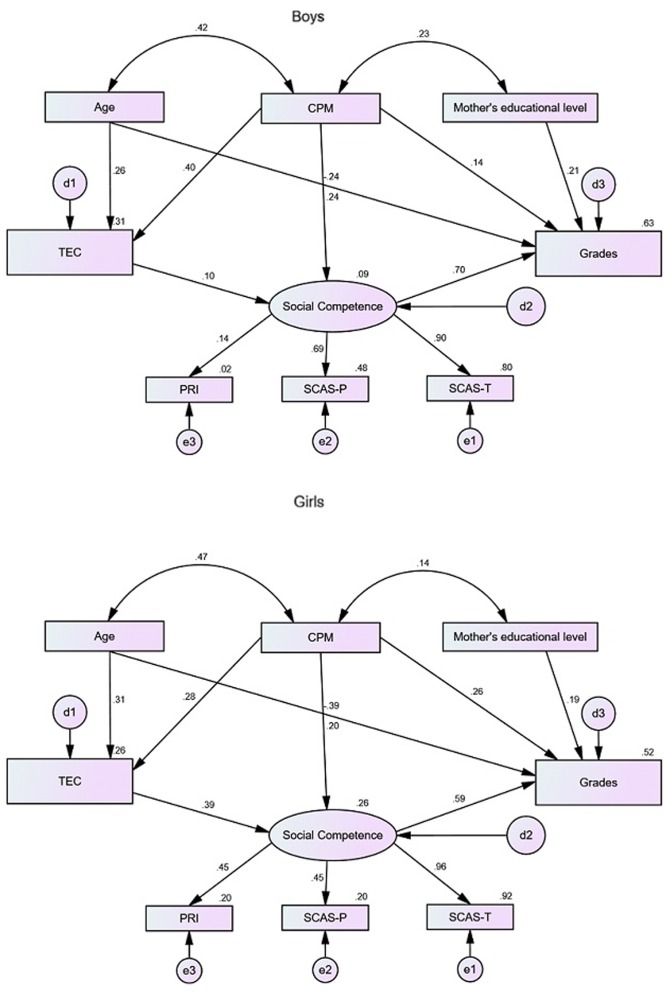
Model 4, causal model of emotion understanding for academic achievement, mediated by social competence, in two student groups (boys and girls) with estimates for structural coefficients, factorials loads and standardized individual reliability (*X*^2^/*df* = 1.359, CFI = 0.974, RMSEA = 0.030, AIC = 156.775).

Lastly, to determine the differences between gender in model 4, path analysis was applied for boys and girls. **Figure 4** illustrates the estimates of the factorial weights and of individual reliability of the items of the model. The quality indexes for the multigroup model were significant: (*X*^2^/*df* = 1.359, CFI = 0.974, TLI = 0.936, RMSEA = 0.030, IC a 90% [0.000;0.051], *p[rmsea]* = 0.940, AIC = 156.775), proving that the proposed factorial model presents a good adjustment, simultaneously for both boys and girls, demonstrating the configurational invariance of the measuring model. The constrict model, with fixed factorial weights, did not present a significantly worse adjustment levels to the model with arbitrary parameters (Δ*X*^2^_λ_(3) = 6.983, *p* = 0.072), in which we may conclude that the measuring model presents low invariance levels, where factorial weights do not differ significantly between boys and girls.

Once the invariance of the measuring model had been settled, it was shown the model that has fixed factorial weights and structural coefficients did not differ significantly to the model with free structural coefficients and fixed factorial weights (Δ*X*^2^_λ_(7) = 10.014, *p* = 0.188). Consequently, the causal model is invariant in both groups, not having been found any evidence of significant differences between boys and girls.

## Discussion

As suggested in the accumulated research in the past years (Izard et al., 2001; Trentacosta and Izard, 2007; Zins et al., 2007; Rhoades et al., 2011; Torres et al., 2015) in this study we found evidence that emotion understanding is a poor predictor of academic achievement. The addition of emotion understanding to the model merely adds to the explained variance value by 1.2%, a modest increase that is consistent with other studies carried out in high school students (Agnoli et al., 2012; Qualter et al., 2012; Downey et al., 2014). Barchard (2007) suggests that is not enough to determine whether emotional competences predict academic achievement, but it’s also necessary to determine whether it betters it’s prediction and if it is worth including emotional competence measurements in existing assessment batteries.

What the findings demonstrate is that there is an interaction between emotion understanding and social competence in the prediction of academic achievement, improving the variance explained by the model in 30.6% and contributing with new findings that establish a positive relationship between social and emotional competences and academic achievements (Caprara et al., 2000; Izard et al., 2001; Denham et al., 2012b; Oberle et al., 2014). Social competence is a mediator of the relationship between emotion understanding and academic achievement, facilitating the relationship with others. In this manner, the predictive ability of social competences, and indirectly of emotion understanding, is significant, even when fluid intelligence and mother’s educational level are monitored.

Social information processing includes competences that require emotion understanding. The ability to understand emotions is related to the communication of the emotion, and when in scarcity, leads to inadequate or insufficient communication, jeopardizing social competence (Machado et al., 2008). Furthermore, the interpretation, modulation and implementation of emotion allow children to respond prosocially in social situations (Mostow et al., 2002; Belacchi and Farina, 2010). In this manner, social competence is set on a series of social, cognitive and emotional abilities of the individual to deal with interpersonal relations that occur in various contexts, encouraging healthier and more beneficial relationships with others (Del Prette and Del Prette, 2005). The ability to establish healthy relationships plays an essential role in human development in general and in particular in school activities, influencing the relationship with teachers, academic achievement and approval among peers (Carrilho, 2012). Social competences are important for efficient learning, according to Elijah and Madeira (2013), because they provide positive relationships between the individual and other social agents, benefitting the participation in classroom activities, for example: exchanging information, requesting orientation or correction, awaiting their turn to speak, follow rules and orientate oneself to the activity (Del Prette and Del Prette, 2005).

As in previous studies (Colom and Flores-Mendoza, 2007; Di Fabio and Palazzeschi, 2009, 2015, Downey et al., 2014) in this study, cognitive abilities significantly predict academic achievement, regardless of the mother’s educational level. The findings also indicate that fluid intelligence predicts emotion understanding and social competences. According to the emotional intelligence ability model, it is expected that emotional competences be moderately correlated to intelligence levels (Garner, 2010; Agnoli et al., 2012; Djambazova-Popordanoska, 2016). In fact, of all emotional abilities, emotion understanding tends to have a stronger correlation with general cognitive functions, with similar values to those found in this study (between 0.40 and 0.60) (Brackett et al., 2011).

The relationship between social competence and fluid intelligence was also expected. Social abilities require some flexibility of thought and of behavior in order to approach and deal with the various social and emotional stimulants. In the processing of social information, emotions and cognitions work together to allow the child to understand and precisely interpret social clues, clarify social objectives, select and implement socially appropriate responses (Mostow et al., 2002). At school, this will be reflected in the facility to transition from one activity to the next in the classroom, not to persist on the same task and try new strategies to complete school activities (Feitosa et al., 2012).

There is evidence that age is a significant predictor of emotion understanding, but not of social competence. In this study, the general scoring in emotion understanding increases regularly with age, as previously found in other studies carried out with TEC (Farina et al., 2007; Albanese et al., 2010; Santos, 2012; Silva, 2012; Franco and Santos, 2015). On the other hand, despite the fact that some studies indicate a connection between age and the development of social competences (Cecconello and Koller, 2000; Major, 2011), in this study these relationships were not found. A possible explanation for these results can be related to the way social competence was assessed, because teachers and parents assess their children’s social competences comparatively to other children of the same age, diluting the effect of age.

As expected, mother’s educational level is a predictor of academic achievement, confirming other studies (Magnuson, 2007; Dubow et al., 2009; Rocha, 2016). In the same manner, no significant effects were found between the mother’s educational level and emotional understanding (Gameiro, 2012; Rocha, 2016) and social competence. Nonetheless, in other studies these variables are related (Alves, 2006; Rhoades et al., 2011; Lima, 2012; Nunes, 2012; Denham et al., 2013; Rocha, 2016).

Lastly, no gender differences were found in the causal model of academic achievement. In some studies gender differences were found regarding academic achievement (Duckworth and Seligman, 2006; Mestre et al., 2006; Deary et al., 2007), emotional competences (Mathieson and Banerjee, 2011; Naghavi and Redzuan, 2011; Denham et al., 2013) and social competences (Denham et al., 2012b, 2013) when studied separately. When these variables were study together the gender differences diminished (Trentacosta and Izard, 2007; Montroy et al., 2014; Rocha, 2016).

In our sample, there is no evidence that gender influences the way social competences mediate the relationship between emotion understanding and academic achievement. A possible explanation suggested by Montroy et al. (2014) is that gender differences tend to appear in older children, of middle and high school. Gender differences in the first years of primary school aren’t always documented (Albanese et al., 2007; Farina et al., 2007; Gustafson, 2009; Matthews et al., 2009; Belacchi and Farina, 2010) but increase in magnitude with age (Montroy et al., 2014). Research carried out with adolescents find greater levels of emotion understanding and social skills among girls than among boys (Welsh et al., 2001; Brackett et al., 2004; Oberle et al., 2014). This may be because as children grow up, intergrupal processes may contribute to the development of preconception and discrimination based on gender (Leaper, 2011). Recent studies show that restrictive gender roles have consequences on girls’ academic achievements (Brown and Leaper, 2010) and on boy’s socioemotional development (Oransky and Fisher, 2009).

In summary, the present study examines the relationship between fluid intelligence, mother’s educational level, gender emotion understanding and social competence as predictors of academic achievement. These findings contribute to the current understanding of the influence of academic achievement, with significant implications to education support and suggest orientations for future studies. Aiding primary school children to achieve academic success it is important not only to promote academic abilities, as well as social and emotional skills (Greenberg et al., 2003; Fleming et al., 2005; Payton et al., 2008; Durlak et al., 2011; Collaborative for Academic, Social, and Emotional Learning [CASEL], 2012).

One of the limitations of this study was the use of grades attributed by the teachers as a measure of academic achievement, because if grades are of easy access through teachers or school archives, their reliability may be limited by the criteria differences among teachers (McMillan, 2001; Oberle et al., 2014). A way to overcome this particular limitation would be to consider different indexes of academic performance simultaneously, and more objective, standardized tests (Di Fabio and Palazzeschi, 2009; Oberle et al., 2014).

In the same manner, personality characteristics were not taken into consideration, which could have been important, seeing that some authors defend that intelligence and personality are better predictors of academic achievement than emotional and social competences (e.g., Barchard, 2007).

Another variable that could be analyzed in future studies is teacher–child relationships. Different studies reveal that teacher–child relationships as well as affective relation between them (Franco, 2012) are important to the comprehension of school achievement (Hamre and Pianta, 2001; Pianta and Stuhlman, 2004; Franco and Beja, 2010; Pasta et al., 2013), school adjustment (Pianta and Steinberg, 1992; Baker, 2006) and the development of social and emotional competences (Birch and Ladd, 1998; Zhang and Nurmi, 2012; Sette et al., 2014; Skalická et al., 2015).

It would also be important to carry out longitudinal studies that look into the predictive role of social and emotional aspects in academic results, during different periods of development (Hawkins et al., 2008; Oberle et al., 2014).

## Ethics Statement

This study was carried out in accordance with the recommendations of Portuguese Psychologists Bar with written informed consent from all subjects. All subjects gave written informed consent in accordance with the Declaration of Helsinki.

## Author Contributions

Conceptualization: MF, NS, and MB; Methodology: MF and NS; Formal Analysis: MF, MB, and AC; Investigation: MF and NS; Writing-Review and Editing: MF, NS, and MB; Supervision: MF, MB, and AC.

## Conflict of Interest Statement

The authors declare that the research was conducted in the absence of any commercial or financial relationships that could be construed as a potential conflict of interest.
